# Impact of climate change on backup energy and storage needs in wind-dominated power systems in Europe

**DOI:** 10.1371/journal.pone.0201457

**Published:** 2018-08-22

**Authors:** Juliane Weber, Jan Wohland, Mark Reyers, Julia Moemken, Charlotte Hoppe, Joaquim G. Pinto, Dirk Witthaut

**Affiliations:** 1 Forschungszentrum Jülich, Institute of Energy and Climate Research – Systems Analysis and Technology Evaluation (IEK-STE), Jülich, Germany; 2 University of Cologne, Institute for Theoretical Physics, Cologne, Germany; 3 University of Cologne, Institute for Geophysics and Meteorology, Cologne, Germany; 4 Karlsruhe Institute of Technology, Institute of Meteorology and Climate Research, Karlsruhe, Germany; 5 Forschungszentrum Jülich, Institute of Energy and Climate Research – Troposphere (IEK-8), Jülich, Germany; 6 University of Cologne, Rhenish Institute for Environmental Research, Cologne, Germany; Centro de Investigacion Cientifica y de Educacion Superior de Ensenada Division de Fisica Aplicada, MEXICO

## Abstract

The high temporal variability of wind power generation represents a major challenge for the realization of a sustainable energy supply. Large backup and storage facilities are necessary to secure the supply in periods of low renewable generation, especially in countries with a high share of renewables. We show that strong climate change is likely to impede the system integration of intermittent wind energy. To this end, we analyze the temporal characteristics of wind power generation based on high-resolution climate projections for Europe and uncover a robust increase of backup energy and storage needs in most of Central, Northern and North-Western Europe. This effect can be traced back to an increase of the likelihood for long periods of low wind generation and an increase in the seasonal wind variability.

## Introduction

The mitigation of climate change requires a fundamental transformation of our energy system. Currently, the generation of electric power with fossil fuel-fired power plants is the largest source of carbon dioxide emissions with a share of approximately 35% of the global emissions [1]. These power plants must be replaced by renewable sources such as wind turbines and solar photovoltaics (PV) within at most two decades to meet the 2°C or even the 1.5°C goal of the Paris agreement [2–4]. While wind and solar power have shown an enormous progress in efficiency and costs [5, 6], the large-scale integration into the electric power system remains a great challenge.

The operation of wind turbines is determined by weather and climate and thus strongly depends on the regional atmospheric conditions. Hence, the generated electric power is strongly fluctuating on different time scales. These fluctuations are crucial for system operation [6–11]. In particular, large storage and backup facilities are needed to guarantee supply also during periods of low wind generation [12–14]. How does climate change affect these fluctuations and the challenges of system integration? Previous studies have addressed the impact of climate change on the availability of cooling water [15, 16], the energy demand [17, 18], the combination of run-of-river and PV [19] or the change of global energy yields of wind and solar power [20–27]. However, the potentially crucial impact of climate change on temporal wind fluctuations has not yet been considered in the literature. In this article, we provide an in-depth analysis of the temporal statistics of wind generation in a changing climate and we assess their potential impact on energy system operation.

A consensus exists about general changes in the mean sea level pressure and circulation patterns in the European/North Atlantic region [28–31]. A projected increase of the winter storminess over Western Europe [32, 33] leads to enhanced wind speeds over Western and Central Europe, while in summer a general decrease is identified [23–25, 34]. This can lead to a strong increase of the seasonal variability of wind power generation and thus impede system integration, even though the annual mean changes are comparatively small.

In this article, we study how climate change affects the temporal characteristics of wind power generation and the necessity for backup and storage infrastructures in wind-dominated power systems in individual European countries and for a perfectly interconnected European power system. Our analysis is based on five state-of-the-art global circulation models (GCMs) downscaled by the EURO-CORDEX initiative [35, 36]. We complement our results with an assessment of the large ensemble of the Coupled Model Intercomparison Project Phase 5 (CMIP5, [37]) based on circulation weather types [38]. The paper is organized as follows. We first introduce our model to derive the backup need of a country as a function of the storage capacity. Additionally, we present the methods to analyze the CMIP5 ensemble. Afterwards we report our results. The article closes with a discussion.

## Methods

**Fig 1:**
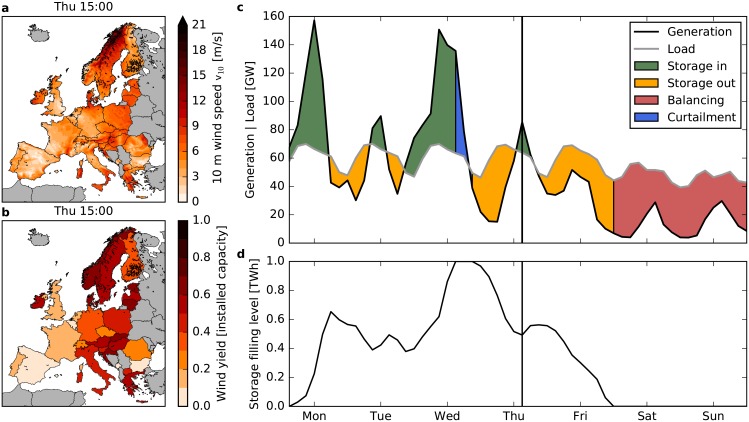
Conversion of near-surface wind speeds to country-wise aggregated wind power generation combined with backup and storage infrastructures. **a**, Near-surface wind velocities of the downscaled ERA-Interim data over Europe for one exemplary point in time. **b**, Corresponding estimated wind power yield for each country in units of the installed capacity at this exemplary time step. **c**, Renewable generation (black) and load (grey) in Germany for one exemplary week in spring assuming a power system with 100% wind power on average. The vertical line denotes the time selected in panels **a** and **b**. The color indicates the operation of the storage system. Green: Excess power is stored. Yellow: Residual load is covered by the storage. Red: Residual load is covered by backup power plants as the storage is empty. Blue: Excess power must be curtailed as the storage is fully charged. **d**, Evolution of the storage filling level *S*(*t*).

The operation of future renewable power systems with large contributions of wind crucially depends on weather and climate. GCMs are used to simulate the dynamics of the earth system on coarse spatial scales for different scenarios of future greenhouse gas concentrations (representative concentration pathways, RCPs [39]). To analyze the operation of the electric power system, a high spatial and temporal resolution is required. Our analysis is thus based on a subset of the EURO-CORDEX ensemble which provides dynamically downscaled climate change data at high resolution (0.11° and 3 hours). Time series for the aggregated wind power generation in a country are obtained from the near-surface wind speed (see Fig 1a and 1b).

Backup and storage infrastructures are needed when renewable generation drops below load. In order to quantify the necessary amount of backup and storage to ensure a stable supply, we adopt a coarse-grained model of the electric power system (see Fig 1c and 1d). Backup and storage needs crucially depend on the temporal characteristics of wind power generation, in particular the length of periods with low wind generation and the seasonal variability. In the present paper, we thus focus on temporal characteristics and their potential alteration due to climate change.

### Wind power generation time series

Our analysis is based on a subset of the EURO-CORDEX regional climate simulations which provides dynamically downscaled climate change data at high resolution for Europe based on five GCMs: CNRM-CM5, EC-EARTH, HadGEM2-ES, IPSL-CM5A-MR, MPI-ESM-LR [35] (see also Table A in S1 Appendix). All data is freely available for example at the ESGF (Earth System Grid Federation) node at DKRZ (German Climate Computing Centre) [40]. The five models are downscaled using the hydrostatic Rossby Centre regional climate model RCA4 [41, 42]. The downscaling provides continuous surface (10 m) wind data from 1970 to 2100 with a spatial resolution of 0.11° and a temporal resolution of *T* = 3 hours. Unfortunately, downscaled data at this high spatial and temporal resolution is not yet available for more GCMs or for different regional climate models at ESGF [40]. Considering the use of only one regional climate model, Moemken *et al.* [25] show that differences between different GCMs are usually larger than differences between different regional climate models.

We analyze a strong climate change scenario (RCP8.5) using a rising radiative forcing pathway leading to additional 8.5 W/m^2^ (∼1370 ppm CO_2_ equivalent) by 2100 and a medium climate change scenario (RCP4.5, ∼650 ppm CO_2_ equivalent, see S3 Appendix) [39]. We compare two future time frames, 2030-2060 (mid century, ‘mc’) and 2070-2100 (end of century, ‘eoc’), to a historical reference time frame (1970-2000, ‘h’).

The calculation of wind power generation requires wind speeds at the hub height of wind turbines. As the high resolution wind velocities are only available at a height of *z*_0_ = 10 m, they must be extrapolated to a higher altitude. We choose a hub height of *z* = 90 m as in [22] and extrapolate the surface wind velocities $v_{z_{0}}$ using a power law formula: $v_{z}=v_{z_{0}}{\left(z/z_{0}\right)}^{1/7}$ [43]. Although widely used, this simple formula is only valid for smooth open terrain and only applies for a neutrally stable atmosphere [44, 45]. Unfortunately, the available data set does not allow to assess the stability of the atmosphere. Thus, it is unclear how to improve the scaling law with the present data available. Tobin *et al.* [22] show in a sensitivity study that their results hardly depend on the extrapolation technique or on the chosen hub height. They further state that the “uncertainty related to climate model formulation prevails largely over uncertainties lying in the methodology used to convert surface wind speed into power output”.

The wind generation is derived using a standardized power curve with a cut-in wind speed of *v*_*i*_ = 3.5 m/s, a rated wind speed of *v*_*r*_ = 12 m/s and a cut-out wind speed of *v*_*o*_ = 25 m/s as in [22]. The capacity factor *CF*(*t*) (i.e. the generation normalized to the rated capacity) then reads

$$\begin{array}{c} CF\left(t\right)=\{\begin{array}{cc} 0 & \text{if}\;v_{z}\left(t\right)<v_{i}\;\text{or}\;v_{z}\left(t\right)\geq v_{o}.\\ \frac{v_{z}^{3}\left(t\right)-v_{i}^{3}}{v_{r}^{3}-v_{i}^{3}} & \text{if}\;v_{i}\leq v_{z}\left(t\right)\leq v_{r}\\ 1 & \text{else}. \end{array} \end{array}$$ (1)

In order to account for wind farms and regional (or sub-cell) velocitiy variations, the power curve is smoothed using a gaussian kernel (see Fig A in S1 Appendix)

$$\begin{array}{c} \text{Ker}\left(v\right)=\frac{1}{\sqrt{2\pi \sigma^{2}}}\exp[\frac{-(v_{0}{-v)}^{2})}{2\sigma^{2}}], \end{array}$$ (2)

where we chose *v*_0_ = *v*_max_/2 + 0.3 m/s and *σ* = 1 m/s with *v*_max_ being the maximum of the occurring wind velocities *v* at hub height. The parameters were chosen such that the rated wind power output is reached for wind speeds which are a little bit higher than the chosen rated wind speed [43, 46].

To obtain the gross generation per country, we equally distribute wind farms on grid points for which the local average wind yield is higher than the country average (see Fig B in S1 Appendix) [47]. The distribution is fixed using historical reanalysis data from ERA-Interim [48] downscaled by the EURO-CORDEX initiative [35, 41] to guarantee consistency (cf. Fig 1b). We do not use the wind farm distribution as of today because installed capacities in a fully-renewable power system will be much higher and also more widespread than they are today such that wind parks will be built in yet unused locations. Furthermore, it was shown in [47, 49] that different wind farm distributions do not significantly affect the results (see also Figs D-F in S4 Appendix where we tested a homogeneous wind farm distribution within each country).

Wind power generation is aggregated using two approaches: (a) aggregation per country neglecting transmission constraints, assuming an unlimited grid within each country; (b) aggregation over the whole European continent, assuming a perfectly interconnected European power system (copperplate). If we find the same results for both cases, we can assume that these results also hold for the intermediate case. The intermediate case is discussed elsewhere [50, 51] for current climatic conditions and in Wohland *et al.* [52] for a changing climate but without considering storage.

As the temporal characteristics of offshore and onshore wind power highly differ from each other, it is important to assess the impact of climate change on offshore and onshore wind separately—at least in a first step. Therefore, in this study, offshore sites are not considered.

For the load time series *L*(*t*) we use data of the year 2015 provided by the European Network of Transmission System Operators for Electricity (ENTSO-E, [53]) and repeat this year 31 times. The load time series have been adapted to the calendars of the individual models, if necessary, by e.g. removing the 31st of a month for HadGEM2-ES, which uses a 30-day calendar, or by constructing an additional day for leap years by repeating the 28th of February. In order to avoid trends in the load timeseries, we consider a single year only. Furthermore, we show in a sensitivity study assuming constant loads that our results dominantly depend on the generation timeseries and are hardly affected by the load time series (see Fig C in S4 Appendix). Throughout all time frames we assume that wind power provides a fixed share *γ* of the load *L*(*t*) per country [12]. Hence, the fluctuating wind power generation *R*(*t*) is scaled such that

$$\begin{array}{c} R\left(t\right)=\gamma \;\frac{CF(t)}{\left\langle CF(t)\right\rangle }\;\left\langle L\left(t\right)\right\rangle , \end{array}$$ (3)

where the brackets denote the average over the respective time frame for a given model. This procedure normalizes out a possible change of the gross wind power yield, and thus allows to isolate the effects of a change in the temporal distribution of the renewable generation. As a consequence, in all considered time frames (historical, mid century and end of century), the total amount of energy generated by wind power plants is the same. Only changes in the temporal aspects such as the duration of low-wind periods or the seasonal wind variability can lead to changes in backup energy and storage needs (see the following sections). For the copperplate assumption, the wind power generation is scaled such that each country provides a fixed share *γ* of the country-specific load. Afterwards, the country-specific wind power generation is summed-up to one aggregated time series. In the main manuscript, we focus on a fully renewable power system per country, i.e., *γ* = 1. Results for different values of *γ* are shown in Figs A and B in S4 Appendix.

### Calculation of backup energy needs

Country-wise aggregated wind generation and load data are used to derive the backup energy need of a country given different storage capacities. At each point of time *t* power generation and consumption of a country must be balanced [12, 50, 54]

$$\begin{array}{c} R(t)+B(t)=\Delta (t)+L(t)+C(t), \end{array}$$ (4)

where *R*(*t*) and *B*(*t*) denote the generation by fluctuating renewables and dispatchable backup generators, respectively, *L*(*t*) is the load and *C*(*t*) denotes curtailment (cf. Fig 1c). Δ(*t*) is the generation (Δ(*t*) < 0) or load (Δ(*t*) > 0) of the storage facilities, such that the storage filling level evolves according to (cf. Fig 1d)

$$\begin{array}{c} S(t+T)=S(t)+\Delta (t)\cdot T. \end{array}$$ (5)

where *T* is the duration of one time step (here: 3 hours). The storage filling level must satisfy 0 ≤ *S*(*t*) ≤ *S*_max_ with *S*_max_ being the storage capacity. We decide to minimize the total backup energy

$$\begin{array}{c} \min B_{\text{tot}}=\sum_{t}B\left(t\right)\cdot T \end{array}$$ (6)

which also minimizes fossil-fuel usage and hence greenhouse gas emissions. One option to minimize *B*_tot_ is to consider a storage-first strategy [54], which we apply sequentially: In the case of overproduction (i.e. *R*(*t*) > *L*(*t*)) excess energy is stored until the storage device is fully charged,

$$\begin{array}{c} \Delta \left(t\right)=\min[R\left(t\right)-L\left(t\right);\left(S_{\text{max}}-S\left(t\right)\right)/T]. \end{array}$$ (7)

To ensure power balance, we may need curtailment

$$\begin{array}{c} C(t)=R(t)-L(t)-\Delta (t). \end{array}$$ (8)

In the case of scarcity (i.e. *R*(*t*) < *L*(*t*)) energy is provided by the storage infrastructures until they are empty,

$$\begin{array}{c} \Delta (t)=-\min[L(t)-R(t);S(t)/T]. \end{array}$$ (9)

The missing energy has to be provided by backup power plants,

$$\begin{array}{c} B(t)=L(t)-R(t)+\Delta (t). \end{array}$$ (10)

The backup power *B* is not restricted in our model and can be interpreted as the aggregated amount of backup power per country, not differentiating between different technologies. In order to keep the storage neutral, a periodic boundary condition is applied [55]: We run the above algorithm twice. First, we choose *S*(*t* = 0) = *S*_max_/2. In the second run, we set *S*(*t* = 0) to *S*(*t* = *t*_max_) of the first run. This way, the storage filling level at *t* = *t*_max_ equals the initial storage filling level at *t* = 0. We emphasize that by the term ‘storage’ we mean storage regardless of the technical realization. Hence, *S*_max_ describes the total accumulated storage capacity, including virtual storage. For simplicity, we neglect losses in the storage process.

In the figures we show the average backup energy per year *E* = 〈*B*〉 / 〈*L*〉 · *L*_year_. *L*_year_ is the average yearly gross electricity demand of the respective country. Thus, *E*/*L*_year_ gives the share of energy that has to be provided by dispatchable backup generators [54].

### Persistence of low wind situations

We measure the probability for long low-wind periods during which a high amount of energy is required from storage devices and backup power plants. Therefore, we identify all periods for which the wind power generation is continuously smaller than average (i.e. *R*(*t*) < 〈*R*〉) and record their duration *τ*. We decided to choose 〈*R*〉 as threshold value because we are interested in long periods of underproduction, which cause the storage to become depleted such that backup energy is required. From the single durations *τ*, we can estimate a probability distribution. Extreme events are quantified by the 95% quantile of the distribution.

### Seasonal wind variability

The wind yield in Europe is usually higher in winter than in summer. An increasing seasonal wind variability would refer to higher wind yields in the winter months and/or lower wind yields in the summer months and would lead to higher backup energy needs during summer.

We define the winter-summer ratio of the country-wise aggregated wind power generation as the ratio of the average winter wind generation 〈*R*〉_DJF_ and the average summer wind generation 〈*R*〉_JJA_:

$$\begin{array}{c} R_{\text{winter}-\text{summer}}=\frac{{\left\langle R\right\rangle }_{\text{DJF}}}{{\left\langle R\right\rangle }_{\text{JJA}}}, \end{array}$$ (11)

with ‘DJF’: December, January, February, and ‘JJA’: June, July, August. 〈*R*〉_DJF_ and 〈*R*〉_JJA_ are the mean generations within a certain time frame (historical, mid century, end of century).

### Analysis of low wind periods using a statistical analysis of a large CMIP5 ensemble

Our analysis is complemented with lower resolution data of 22 GCMs contributing to the Coupled Model Intercomparison Project Phase 5 (CMIP5, [37]). The GCM output is analyzed with a statistical method developed by Reyers *et al.* [23, 24]. We characterize the large-scale circulation over Central Europe by determining the prevalent circulation weather type (CWT, [38]) using instantaneous daily mean sea level pressure (MSLP) fields around a central point at 10°E and 50°N (near Frankfurt, Germany) at 00 UTC (see also Fig 2 in Reyers *et al.* [23]). The different CWT classes are either directional (‘North’, ‘North-East’, ‘East’, ‘South-East’, ‘South’, ‘South-West’, ‘West’, ‘North-West’) or rotating (‘Cyclonic’, ‘Anti-cyclonic’). Additionally, a proxy for the large-scale geostrophic wind (denoted as *f*-parameter) is derived using the gradient of the instantaneous MSLP field. Higher geostrophic wind values (i.e. higher *f*-parameters) correspond to larger wind power yields in Central Europe [56].

In order to compare the CMIP5 and the EURO-CORDEX data, we test whether the *f*-parameter derived using the coarse ERA-Interim reanalysis data [23] is capable to reproduce the characteristics of German low-wind generation periods as determined from the downscaled ERA-Interim dataset [48]. We classify days with below-average wind power generation (scarcity) for each CWT by a low value of the *f*-parameter, *f*(*t*) ≤ *f*_th_. Thus, for each day, we can analyze whether the classifier (*f*(*t*) ≤ *f*_th_) correctly predicts that the wind power generation is below average (*R*(*t*) < 〈*R*〉) or erroneously predicts that the wind power generation is above average (*R*(*t*) ≥ 〈*R*〉). The quality of this classification is quantified by the fraction of true predictions, called sensitivity

$$\begin{array}{c} \text{SEN}=\frac{n[R<\left\langle R\right\rangle \;\&\;f\leq f_{\text{th}}]}{n\left[R<\left\langle R\right\rangle \;\&\;f\leq f_{\text{th}}\right]+n\left[R<\left\langle R\right\rangle \;\&\;f>f_{\text{th}}\right]} \end{array}$$ (12)

and the fraction of false predictions

$$\begin{array}{c} \text{FFP}=\frac{n[R\geq \left\langle R\right\rangle \;\&\;f\leq f_{\text{th}}]}{n\left[R\geq \left\langle R\right\rangle \;\&\;f\leq f_{\text{th}}\right]+n\left[R\geq \left\langle R\right\rangle \;\&\;f>f_{\text{th}}\right]}, \end{array}$$ (13)

where *n* denotes the number of days where the conditions are satisfied [57]. A compromise must be found between a maximum sensitivity for high values of *f*_th_ and a minimum fraction of false predictions for low values of *f*_th_. A common choice is to choose the value *f*_th_ which minimizes (1 − SEN)^2^ + FFP^2^ [57] (see also Fig C in S1 Appendix (ROC-curve)). Under the assumption that the meaning of the *f*-parameter does not depend on GCM and time frame, we use the derived *f*_th_ to estimate the duration of low wind periods as described in above.

## Results

### Increase of backup and storage needs

We assess the impact of climate change on the average backup energy per year *E* as a function of the storage capacity *S*_max_. The storage capacity is given in units of the yearly load *L*_year_ of a country and is shown on a range between *S*_max_ = 10^−5^ to *S*_max_ = 10^−1^. The case of *S*_max_ = 10^−5^ can be regarded as the no-storage case. Results hardly differ for even smaller storage capacities. It should be noted that storage capacities above about 10^−3^ correspond to a scenario with massive extension of (effective) storage capacities. This could include the large-scale deployment of novel technologies, in particular chemical storage and/or virtual storage. As in highly renewable power systems huge amounts of storage will be necessary (see e.g. [54, 55]), we decided to consider also these highly optimistic cases.

**Fig 2:**
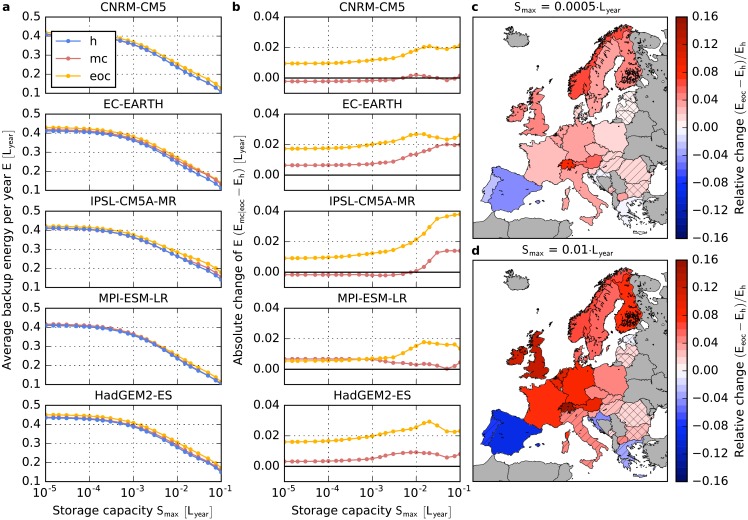
Impact of strong climate change on backup energy needs in Europe. **a,** Amount of energy that has to be provided by dispatchable backup generators in Germany as a function of the storage capacity *S*_max_ for the five models in the EURO-CORDEX ensemble and a strong climate change scenario (RCP8.5). Energy is given in units of the average yearly gross electricity consumption *L*_year_. Blue: 1970-2000 (h), Red: 2030-2060 (mc), Yellow: 2070-2100 (eoc). **b,** Absolute change of the average backup energy as a function of *S*_max_ in Germany. Colors are the same as in panel **a**. **c, d,** Relative change of the average backup energy needs by the end of the century with respect to the historical time frame for 29 European countries and two values of the storage capacity *S*_max_. The color code corresponds to the average of the five models and the hatching indicates the robustness of the results. No hatching: 5/5, striped: 4/5, crossbred: 3/5 models agree on the sign of change.

All models in the EURO-CORDEX ensemble predict an increase of the necessary backup energy in most of Central Europe (i.e. Germany, Poland, Czech Republic, Switzerland, Austria, the Netherlands and Belgium), France, the British Isles and Scandinavia for a strong climate change scenario (RCP8.5) by the end of the century relative to the historical time frame (see Fig 2c and 2d). This implies that even though the same amount of energy is produced by renewables in both time frames, less renewable energy can actually be used. Relative changes are highest in Switzerland and the United Kingdom with a range between 12.2 to 24.2% (ensemble mean: 15.6%) and 7.1 to 16.5% (ensemble mean: 12.1%), respectively for a storage capacity of *S*_max_ = 0.01 · *L*_year_. However, results for mountainous regions like Switzerland should be regarded with caution as wind farms might be placed at sites which are unsuitable. In addition, climate model results over complex terrain are known to have large uncertainties. An opposite effect is observed for the Iberian Peninsula, Greece and Croatia where the need for backup energy decreases (e.g., Spain: -4.7 to -15.5%; ensemble mean: -9.1% for *S*_max_ = 0.01 · *L*_year_). These results hold for a variety of scenarios for the development of storage infrastructures leading to different values of the storage size *S*_max_, being more pronounced for larger storage sizes. The latter partly results from a change in the seasonal variability of the wind power generation (cf. below). In the Baltic region and South-Eastern Europe, relative changes are weaker and the models most often do not agree on the sign of change and can therefore be regarded as not robust [58, 59].

**Fig 3:**
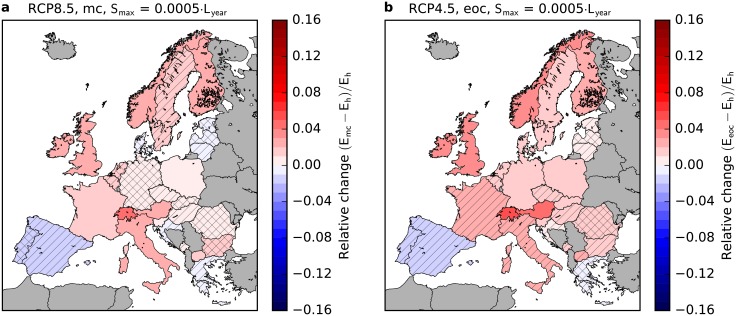
Impact of climate change on backup energy needs in Europe for different time frames and scenarios. a, mid century and strong climate change (RCP8.5). b, end of century and medium climate change (RCP4.5). Further parameters and presentation as in Fig 2c.

Similar changes are observed already at mid century (2030-2060, see Fig 3a and Fig A in S2 Appendix) and for RCP4.5 (see Fig 3b and Fig A in S3 Appendix). However, the results are less pronounced and often not robust.

For Germany (Fig 2a and 2b), the absolute increase of the average backup energy per year *E* amounts to 0.6-3.8% of the average yearly consumption *L*_year_ by the end of the century. Assuming a yearly consumption of the order of *L*_year_ = 600 TWh [60], this corresponds to an additional need of 4-23 TWh of backup energy per year.

**Fig 4:**
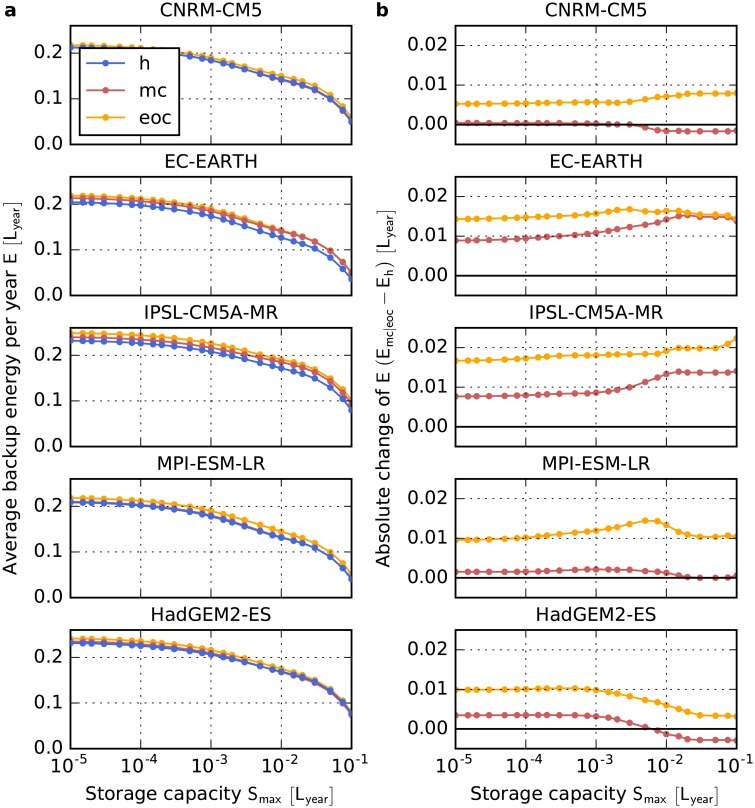
Impact of strong climate change on backup energy needs for a perfectly interconnected European power system. **a,** Amount of energy that has to be provided by dispatchable backup generators in Europe as a function of the storage capacity *S*_max_ for the five models in the EURO-CORDEX ensemble and a strong climate change scenario (RCP8.5). Energy is given in units of the average yearly gross electricity consumption *L*_year_. Blue: 1970-2000 (h), Red: 2030-2060 (mc), Yellow: 2070-2100 (eoc). **b,** Absolute change of the average backup energy as a function of *S*_max_. Colors are the same as in panel **a**.

In a perfectly interconnected Europe, the average relative backup energy per year is much smaller than for individual countries (e.g., for Germany, cf Figs 2a and 4a). This is because the balancing takes place over a large spatial scale with many different wind patterns at the same time step. For all five models and all storage capacities, we find an increase of the average backup energy per year *E* by the end of the century (see Fig 4). Values range from 0.3 to 2.2% of the average yearly consumption *L*_year_. For high storage capacities, the change depends strongly on the seasonal wind variability (cf. below). Hence, we find increasing backup energy needs for many single European countries as well as for a perfectly interconnected Europe. This implies that, even though balancing takes place over large spatial scales, certain wind situations occur simultaneously in many countries. In fact, Wohland *et al.* [52] find that wind conditions become more homogeneous within Europe in a future climate, which decreases inter-state balancing of electricity. For mid century, the same effect albeit at a weaker magnitude can be observed.

Two main drivers for the increase in the backup energy can be identified: a higher probability for long periods with low wind power generation and a higher seasonal wind variability.

### Challenges by long low-wind periods

**Fig 5:**
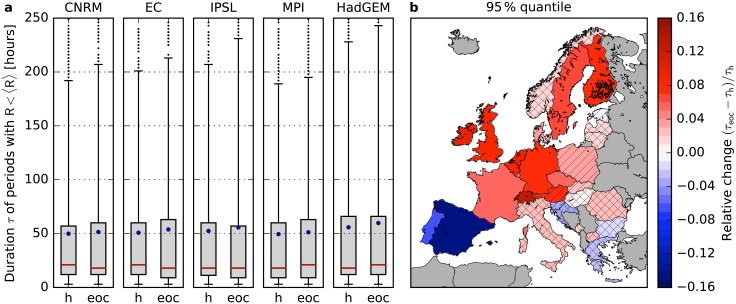
Change of the duration of periods with low wind generation. **a**, Distribution of the duration of periods during which the wind generation is continuously lower than average (*R*(*t*) < 〈*R*〉) in Germany for the five models in the EURO-CORDEX ensemble. Boxes represent the 25% to 75% quantiles, whiskers indicate the 5% and 95% quantiles, the red line is the median, the blue dot shows the mean and black dots represent outliers. Results are shown for the historical time frame (h, 1970-2000) and the end of the century (eoc, 2070-2100) for a strong climate change scenario (RCP8.5). **b**, Relative change of the duration assigned to the 95% quantile by the end of the century with respect to the historical time frame for 29 European countries. The color code corresponds to the average of the five models and the hatching indicates the robustness of the results. No hatching: 5/5, striped: 4/5, crossbred: 3/5 models agree on the sign of change.

During long periods of low renewable generation, the storage facilities get depleted with a high probability such that the residual load has to be covered by backup power plants leading to a high backup energy need. In Fig 5 we show the duration distribution of periods for which wind power generation is continuously lower than average (i.e. *R*(*t*) < 〈*R*〉) for Germany (panel a) and the relative change of the 95% quantile (panel b). The 95% quantile shifts to longer durations in most of Central Europe, France, the British Isles, Sweden and Finland and decreases on the Iberian Peninsula by the end of the century. These findings are robust in the sense that all five models in the EURO-CORDEX ensemble agree on the sign of change as illustrated for Germany in Fig 5a.

Long low-wind periods are crucially difficult for the operation of future renewable power systems [13]. An increasing magnitude for such extreme events thus represents a serious challenge for renewable integration. In Eastern Europe, Italy, Greece and Norway relative changes are weaker and not robust. The effect develops mostly in the second half of the century (cf. Fig B in S2 Appendix) and for strong climate change (RCP8.5, cf. Fig B in S3 Appendix).

**Fig 6:**
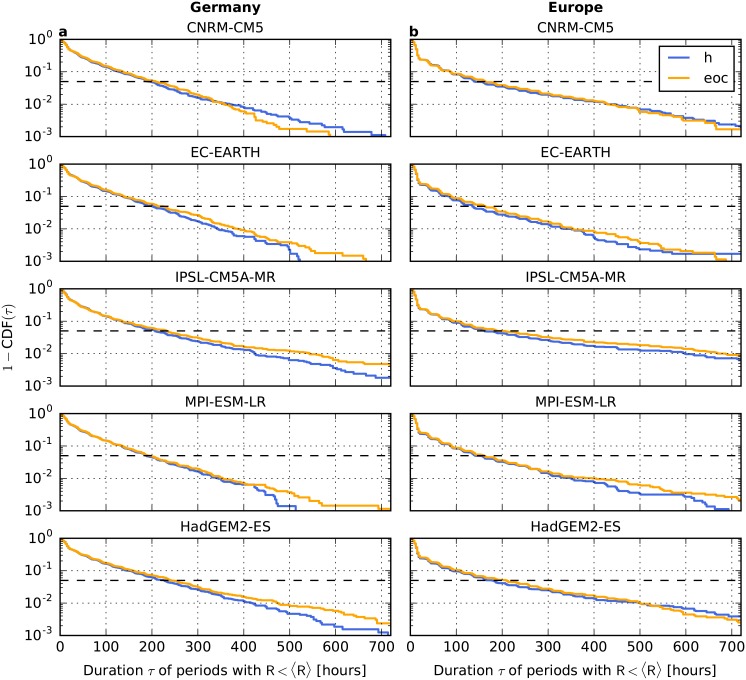
Change of the duration of periods with low wind generation. **a,** One minus the cumulative distribution function (CDF) of periods having a duration *τ* during which the wind generation is continuously lower than average (*R*(*t*) < 〈*R*〉) for Germany and **b,** for the European copperplate. Results are shown for the historical time frame (h, 1970-2000) and the end of the century (eoc, 2070-2100) for a strong climate change scenario (RCP8.5) for the five models in the EURO-CORDEX ensemble. The dashed vertical line represents the 95%-quantile of the CDF.

The complete distribution of durations is shown in Fig 6. We find that for Germany (panels a) not only the duration associated with the 95%-quantile tends to increase but also the probability for particularly long durations (except for CNRM-CM5). Considering the perfectly interconnected European power system (panels b), we also find that the 95% quantile shifts to higher values by the end of the century. Furthermore, the probability for low-wind periods having a duration of up to about 500 hours increases (again except for CNRM-CM5). For longer durations, the curves often cross. However, it is difficult to evaluate such extreme events appropriately in the context of climate change given the finite duration of the time series. All in all, this analyis indicates that long lasting low-wind conditions, which extend over the whole European continent, are projected to become more likely (see also [52]).

### Higher seasonal wind variability

The second reason for an increase of backup and storage needs is an increasing intensity of the seasonal wind variability. Typically, the wind power yield is highest in the winter months such that backup power plants are needed mostly in summer.

**Fig 7:**
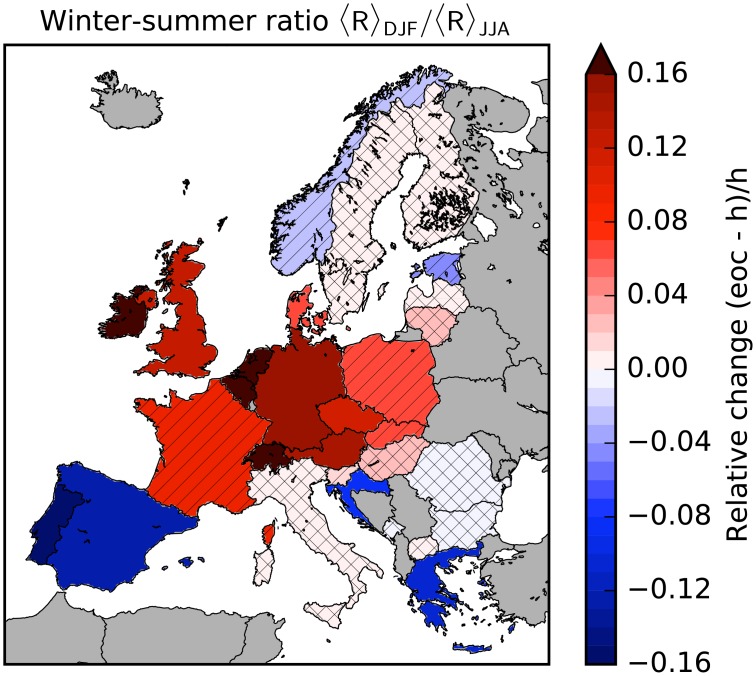
Impact of strong climate change on the seasonal variability of wind power generation. Relative change of the winter-summer ratio of the average wind power yield 〈*R*〉_DJF_/〈*R*〉_JJA_ (DJF: December-February vs. JJA: June-August) by the end of the century (eoc, 2070-2100) with respect to the historical time frame (h, 1970-2000). The brackets denote the temporal average over the respective winter and summer months. Results are shown for a strong climate change scenario (RCP8.5) for 29 European countries. The color code corresponds to the average of the five models and the hatching indicates the robustness of the results. No hatching: 5/5, striped: 4/5, crossbred: 3/5 models agree on the sign of change.

The winter-summer ratio increases for most of Central and North-Western Europe, and decreases for the Iberian Peninsula, Greece and Croatia (see Fig 7) for four or all five models in the EURO-CORDEX ensemble. In these countries the seasonal variability therefore contributes to the observed changes of backup needs. Changes are small and not robust in Italy, most of Eastern Europe and Scandinavia (except Denmark). Hence, the increase of backup needs in Northern Europe is attributed solely to the higher probability for long periods with low wind power generation. For mid century (see Fig C in S2 Appendix), and for medium climate change (RCP4.5, see Fig C in S3 Appendix), results are comparable but less robust for some countries.

For the perfectly interconnected European power system, four of the five models predict an increasing seasonal wind variability in the range of 4.1 to 10.4%. Thus, the lower seasonal wind variability on the Iberian Peninsula, Greece and Croatia cannot totally compensate the higher seasonal wind variability in the other European countries. In contrast, HadGEM2-ES predicts a decrease of -2.8%.

The higher seasonal wind variability also explains the relative increase of the backup energy for higher storage capacities (cf. Fig 2). A high storage capacity allows to store some part of the energy for several months. However, as the storage capacity is still limited, a higher seasonal wind variability implies that the storage is fully charged earlier in winter and that it is depleted earlier in summer. Thus, less excess energy can be transferred from the winter to the summer months if the seasonal variability of wind power generation increases.

The duration of low-wind periods is strongly associated with the seasonal wind variability, e.g. low-wind periods are more frequent over Western Europe in summer than in winter. To assess the implications of this connection in a changing climate, we evaluated the distribution of durations of low-wind periods also per season, and computed the changes between the distributions for the end of the century vs. recent climate conditions. We found that in countries where the seasonal wind variability increases, the duration of low-wind periods also increases (in most cases) in all seasons, but primarily in summer. This result is robust for all five models. The same effect, albeit in the reverse direction, is observed for e.g. Spain, where a decreasing duration of low-wind periods is coupled to the decreasing seasonal wind variability and hence to shorter durations of low-wind periods in summer. We note that there are also countries where the probability for long low-wind periods increases, but no change in the seasonal wind variability is observed (e.g. Finland). Hence, we conclude that the change of seasonality and duration are indeed highly coupled, but one effect is not simply the consequence of the other.

### Low-wind periods in a large CMIP5 ensemble

To substantiate our findings, we analyze a large CMIP5 ensemble [37] consisting of 22 GCMs with a much coarser resolution than the EURO-CORDEX ensemble as explained in the methods section.

**Fig 8:**
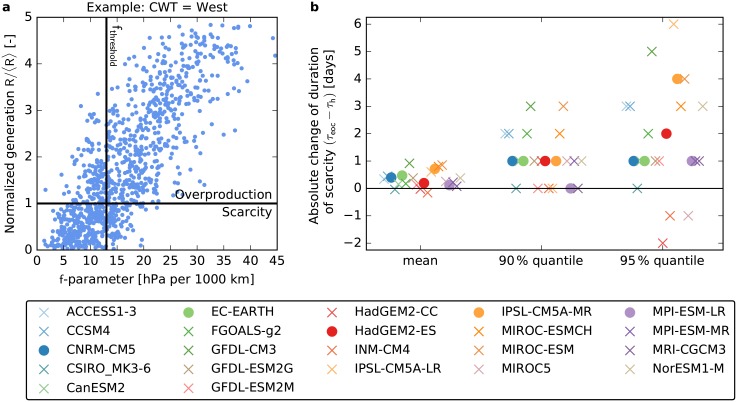
Assessment of long low-wind periods in a large CMIP5 ensemble. **a,** Days with below average wind power generation in Central Europe are identified by a low value of the *f*-parameter (*f*(*t*) ≤ *f*_th_) in the GCM output. To determine the optimal value of the threshold *f*_th_, for each circulation weather type (CWT, here, the western type is shown) we compare the *f*-parameter to the German wind power output *R*(*t*), calculated from the dynamically downscaled ERA-Interim reanalysis dataset [48]. **b,** Absolute change of the duration of periods with *f*(*t*) ≤ *f*_th_ by the end of the century (eoc, 2070-2100) compared to the historical time frame (h, 1970-2000) for a strong climate change scenario (RCP8.5) for 22 GCMs in the CMIP5 ensemble. The change of the mean duration, the 90% quantile and the 95% quantile of the duration distribution are shown. Filled circles represent the five GCMs which are also downscaled by the EURO-CORDEX initiative.

The typical duration of periods with *f*(*t*) ≤ *f*_th_ in Central Europe increases by the end of the century for most GCMs in the CMIP5 ensemble. 19 of the 22 models predict an increase of the mean duration (Fig 8b). The 90% quantile of the duration increases for 16 models and remains unchanged for the remaining six models, while the 95% quantile increases for 18 of the 22 models. Fig 8b shows that the five models of the EURO-CORDEX ensemble (shown as filled circles) form a representative subset of the CMIP5 ensemble since their results are well distributed within the range of the majority of all models and thus do not contain outliers. Hence, the large CMIP5 ensemble corroborates our previous findings, predicting an increase of the likelihood for long periods with low wind power output for a strong climate change scenario.

To assess the sensitivity of the choice of *f*_th_, we repeated our analysis by determining one value for *f*_th_ which is independent of the underlying CWT. This does not change the results as shown in Fig I in S4 Appendix.

### Climatologic developments driving enhanced seasonality

The identified increase in the seasonal variability of wind power generation has been discussed in terms of the projected changes of large-scale atmospheric circulation and regional wind conditions. A consensus exists about general changes in the large-scale circulation patterns in the Eastern North Atlantic region and Europe, which is however dependent on the time of the year [28]. During winter, the eddy-driven jet stream and cyclone intensity are extended towards the British Isles [29]. Accordingly, winter storminess is projected to increase over Western Europe [32, 33], leading to enhanced winds over Western and Central Europe. The signal in summer corresponds rather to a northward shift of the eddy driven jet stream, cyclone activity and lower tropospheric winds, together with an increase in anticyclonic circulation over Southern Europe [30]. The latter is associated with an expansion of the Hadley circulation due to enhanced radiative forcing [31]. These developments are projected to decrease wind speeds during summer [23–25, 34].

These seasonal changes have strong implications not only on temperature and precipitation patterns, but also in the seasonal wind regimes and intra-annual variability. The seasonal variability of wind power generation increases under future climate conditions [23, 25, 34] even though the annual mean changes are comparatively small [21, 22, 24, 25, 34]. The impact may be large for the operational systems, and thus needs to be quantified adequately based on state-of-the-art climate model projections.

## Discussion

Wind power, PV and other renewable sources can satisfy the majority of the global energy demand [5, 6, 61]. However, system integration remains a huge challenge: The operation of wind turbines and PV relies on weather and climate and thus shows strong temporal fluctuations [7–10, 12–14, 51, 62]. The impact of climate change on the global energy yields of wind and solar power has been addressed previously [21–26], but the impact on fluctuations and system integration has been addressed only recently [52, 63, 64].

In this paper, we analyzed the change of the temporal characteristics of wind power generation in a strong (RCP8.5) and a medium climate change scenario (RCP4.5, see Fig 3a and S3 Appendix). Backup and storage needs increase in most of Central, Northern and North-Western Europe and decrease over the Iberian Peninsula, Greece and Croatia. As these effects are observed for both aggregation approaches used in this study (approach (a): aggregation per country, approach (b): European copperplate), we hypothesize that the effect will also be observed in intermediate scenarios with restricted interconnection between countries. By mid century and for medium climate change, results are less pronounced and often not robust. Two main climatologic reasons for the observed increase were identified: a higher probability for long periods of low wind power generation and a stronger seasonal wind variability.

Wohland *et al.* [52] examined climate change impacts for different levels of European grid integration. Since the same climatic input data was used, their results can complement the interpretation of the findings in the current study. Neglecting energy storage, they report an increase of backup energy irrespective of the grid design by the end of the century and RCP8.5.

The projected increase in backup energy needs may partly be compensated in some countries by using an appropriate mix of wind and PV (see also Fig H in S4 Appendix). Furthermore, wind generation from offshore wind farms is often more persistent and installed capacities are strongly increasing. In a further study, climate projections for onshore and offshore wind and PV should thus be analyzed together in order to account for possible changes in the temporal variations of the combined system of renewables.

To isolate the change of the temporal characteristics of wind power generation, we made several simplifications. First of all, we assumed that wind provides a fixed share *γ* of the load for all time frames. This procedure normalizes out a possible change of global wind yields (previously discussed [21–25]). In S4 Appendix Figs A-C, we evaluated the impact of a higher or lower renewable penetration *γ* and of the exact load time series on our results and found the same tendencies, albeit at different magnitude. Technological progress of the wind turbines and changes of typical hub heights were not considered in a detailed way. However, a different siting of wind farms or a higher hub height of 120 m hardly impacts our results (see Figs D-G in S4 Appendix). For an integrated assessment, technological progress should be taken into account, but our approach reveals the impact of climate change on the temporal characteristics clearly.

A reliable interpretation of climate projections should be based on multi-model ensembles [22, 37]. Our analysis of the small EURO-CORDEX ensemble consisting of five models shows robust results regarding the sign of change for several regions in Europe. A statistical analysis of the output of 22 GCMs from the CMIP5 ensemble supports our findings, as the duration of periods with low values of the *f*-parameter over Central Europe is likely to increase. Large-scale climatologic developments leading to an increase of the seasonal wind variability were previously discussed [23, 25, 28–34]. For future research, it would be highly desirable if larger ensembles of dynamically downscaled models would be provided. Furthermore, data at turbine hub height should be made available. Ongoing downscaling experiments within the new CMIP6 CORDEX initiative [65] will allow to assess the impact of climate change on system integration of intermittent renewables for various regions in the same manner. This should include a detailed and explicit analysis on the projected changes of both wind and PV. In conclusion, our work contributes to highlight the importance of integrated energy and climate research to enable a sustainable energy transition.

## Supporting information

S1 Appendix Supporting figures and tables for the methods section. (PDF)

S2 Appendix Mid century (2030-2060). (PDF)

S3 Appendix RCP4.5. (PDF)

S4 Appendix Sensitivity studies. (PDF)
