# Supplementary material for: Impact of climate change on backup energy and storage needs in wind-dominated power systems in Europe
Source: PLoS One. 2018 Aug 22;13(8):e0201457. doi: 10.1371/journal.pone.0201457 (PMC6104926; doi:10.1371/journal.pone.0201457)
Supplement: S1 Appendix — (PDF) [file pone.0201457.s001.pdf]

Appendix 1: Supporting figures and tables for the methods section

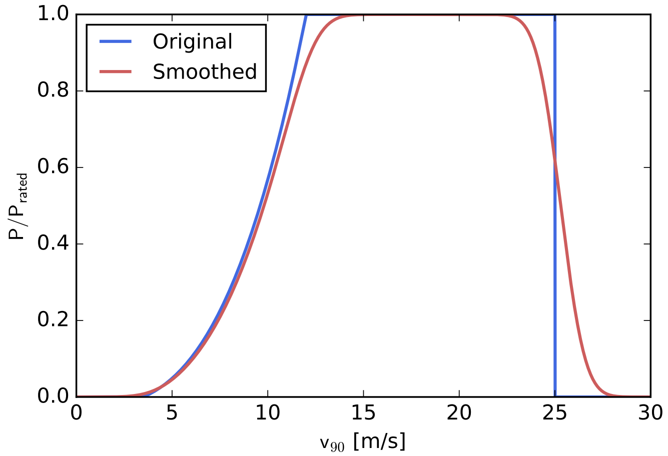

Fig A. Power curve. Power curve used to convert wind velocities in a height of 90 m (v_{90}) to wind power generation data. In order to account for wind farms and velocity variations, the single turbine power curve (blue) is smoothed using a gaussian kernel (red).

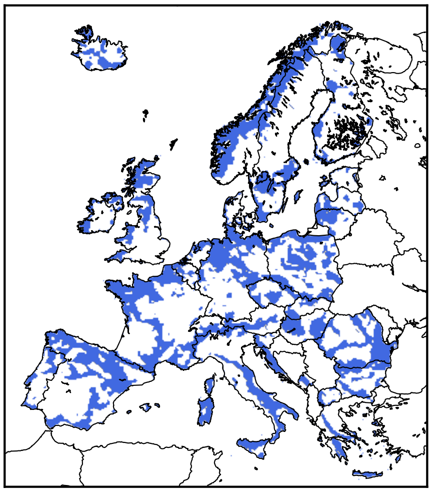

Fig B. Placement of wind farms. Wind farms are homogeneously placed on colored sites. On these sites the 31-year average of the wind yield is higher than the country average. For this derivation, ERA-Interim data [1] is used. In a sensitivity study, wind farms are placed homogeneously at each grid point inside a country (see Figs D-F in S4 Appendix).

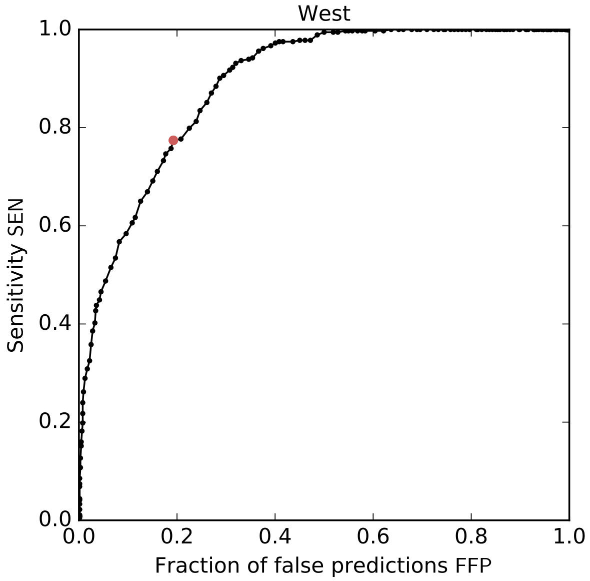

Fig C. ROC curve. Receiver operating characteristic (ROC) curve to assess the performance of the f-parameter to classify days with low wind power generation. For each pre-defined threshold value f_{th}, the sensitivity SEN and the fraction of false predictions FFP are calculated. The optimal value of f_{th} (red dot) minimizes the distance to the point (SEN, FFP) = (1,0), which corresponds to a perfect classifier. Results are shown for the western CWT.

Table A. Global circulation models used in the EURO-CORDEX ensemble. The downscaled data has a resolution of 0.11° and 3 hours [2]. Table adapted from [3]

**Model name**	**Institution**
CNRM-CM5 (CNRM Coupled Global Climate Model, version 5)	Centre National de Recherches Météorologiques (CNRM), France
EC-EARTH (EC-Earth Consortium)	European Consortium (EC)
IPSL-CM5A-MR (IPSL Coupled Model, version 5, coupled with NEMO, medium resolution)	Institut Pierre Simon Laplace (IPSL), France
MPI-ESM-LR (MPI Earth System Model, low resolution)	Max Planck Institute (MPI) for Meteorology, Germany
HadGEM2-ES (Hadley Centre Global Environment Model, version 2, Earth System)	Met Office Hadley Centre, United Kingdom

References

1. Dee D, Uppala S, Simmons A, Berrisford P, Poli P, Kobayashi S, et al. The ERA-Interim reanalysis: Configuration and performance of the data assimilation system. Quarterly Journal of the royal meteorological society. 2011;137(656):553–597.

2. Jacob D, Petersen J, Eggert B, Alias A, Christensen OB, Bouwer LM, et al. EURO-CORDEX: new high-resolution climate change projections for European impact research. Regional Environmental Change. 2014;14(2):563–578. doi:10.1007/s10113-013-0499-2.

3. Reyers M, Moemken J, Pinto JG. Future changes of wind energy potentials over Europe in a large CMIP5 multi-model ensemble. International Journal of Climatology. 2016;36(2):783–796.
