# Supplementary material for: Impact of climate change on backup energy and storage needs in wind-dominated power systems in Europe
Source: PLoS One. 2018 Aug 22;13(8):e0201457. doi: 10.1371/journal.pone.0201457 (PMC6104926; doi:10.1371/journal.pone.0201457)
Supplement: S2 Appendix — (PDF) [file pone.0201457.s002.pdf]

Appendix 2: Mid century (2030-2060)

In the main manuscript, most results are only shown for the end of the century (2070-2100). Here, results shall be shown for mid century (2030-2060).

In Fig A the relative change of the backup energy is shown for two different storage capacities. For most of Central Europe, France, the British Isles, Scandinavia (except Denmark) and Italy four to five models in the EURO-CORDEX ensemble predict an increasing backup need. Exceptions are Germany, where results are not robust for small $S_{\max}$ and Norway, Sweden, Poland and the Czech Republic, where results are not robust for high $S_{\max}$. For Spain, Greece and Croatia (and Portugal for high $S_{\max}$) four models predict a slightly decreasing backup need. Compared to the results we have for the end of the century (cf. Fig 2c and d in the main manuscript), changes are weaker and, therefore, in some countries less robust. For most countries in Eastern Europe results are not robust which is also observed for the end of the century.

The duration distribution of periods with $R(t) < \langle R \rangle$ and the relative change of the 95 % quantile of this distribution are shown in Fig B. In almost all European countries the five models do not agree on the sign of change. These results indicate that changes in the backup energy need can mostly not be attributed to a change in the duration of long low wind periods.

Instead, higher/lower backup needs can be explained by the winter-summer ratio (see Fig C) which is predicted to increase in France, the British Isles, and most of Central Europe (except Germany) by four to five models. Furthermore, the ratio is predicted to decrease on the Iberian Peninsula, Greece and (not robustly) in the Baltic countries. The relative change, however, is lower than by the end of the century (cf. Fig 7 in the main manuscript).

Hence, by mid century, there is still a trend for increasing backup and storage needs due to a change in the seasonal wind variability. However, relative changes are weaker and results are less robust for some countries.

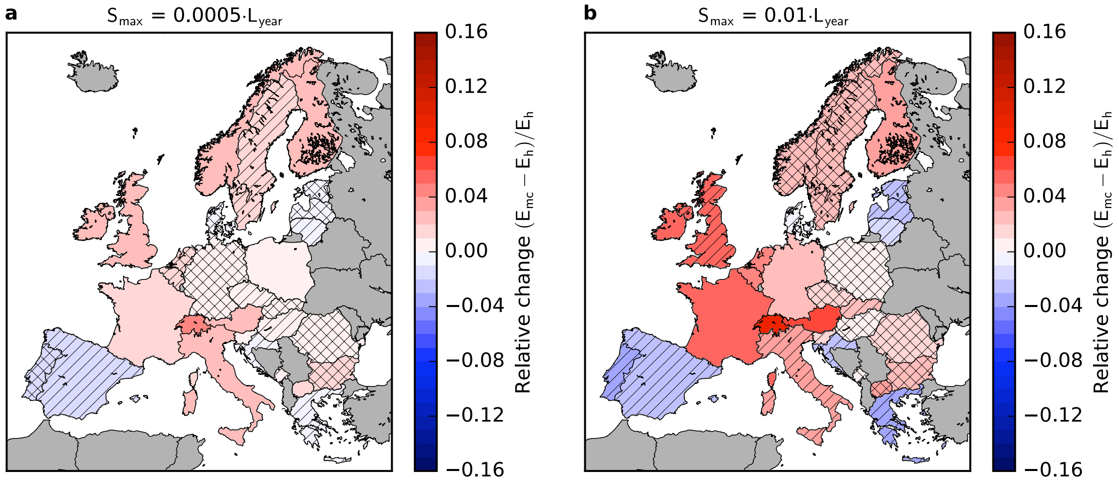

Fig A. Impact of strong climate change on backup energy needs by mid century. Parameters and presentation as in Fig 2.

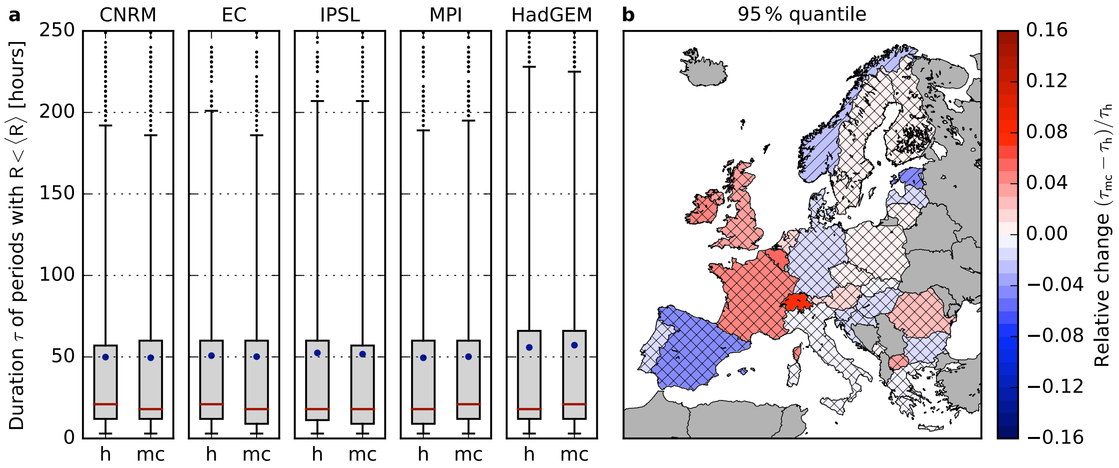

Fig B. Change of the duration of periods with low wind generation by mid century. Parameters and presentation as in Fig 5.

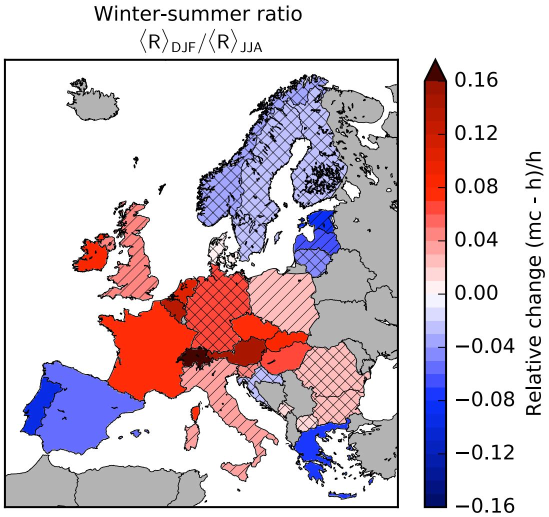

Fig C. Impact of strong climate change on the seasonal variability of wind power generation by mid century. Parameters and presentation as in Fig 7.
