# Supplementary material for: Impact of climate change on backup energy and storage needs in wind-dominated power systems in Europe
Source: PLoS One. 2018 Aug 22;13(8):e0201457. doi: 10.1371/journal.pone.0201457 (PMC6104926; doi:10.1371/journal.pone.0201457)
Supplement: S3 Appendix — (PDF) [file pone.0201457.s003.pdf]

Appendix 3: RCP4.5

We repeated the analysis for the medium climate change scenario RCP4.5 which is leading to an increase of 4.5 W/m^2 in radiative forcing (∼650 ppm CO_2 equivalent) by 2100 [1]. Fig A shows the impact of this scenario on backup energy needs in Germany (panels a and b) and Europe (panels c and d) by the end of the century (2070-2100). The backup need increases in most of Central Europe, France and the British Isles. Even though the increase is weaker than in the RCP8.5 scenario (cf. Fig 2 in the main manuscript), it is important to note that the effect on the backup need is still pronounced in most of these countries. For France, Belgium, Scandinavia, Italy and Poland, the robustness of the results depends on the storage size. As for RCP8.5, results are not robust in most of Eastern Europe. The decrease in the backup need on the Iberian Peninsula (for high $S_{\max}$), Greece and Croatia is not robust.

The increase in the backup need can only partly be explained by an increase in the duration of long low wind periods (see Fig B). Only in Switzerland and Belgium all models agree on the sign of change and on the British Isles, France, Austria and the Czech Republic, four of the five models agree on the sign of change. In the other countries, results are not robust and/or relative changes are small.

Changes in the backup need can mostly be explained by changes in the winter-summer ratio (see Fig C): For those countries, in which the backup energy need increases, the winter-summer ratio also increases (the British Isles, Benelux, France, Switzerland, Austria, Czech, Slovakia, Slovenia, Germany and Italy).

In conclusion, by the end of the century similar trends as in the RCP8.5 scenario are found using the RCP4.5 scenario. However, changes are weaker and, therefore, often less robust. The decreasing backup needs on the Iberian Peninsula, Greece and Croatia are not robust for medium climate change.

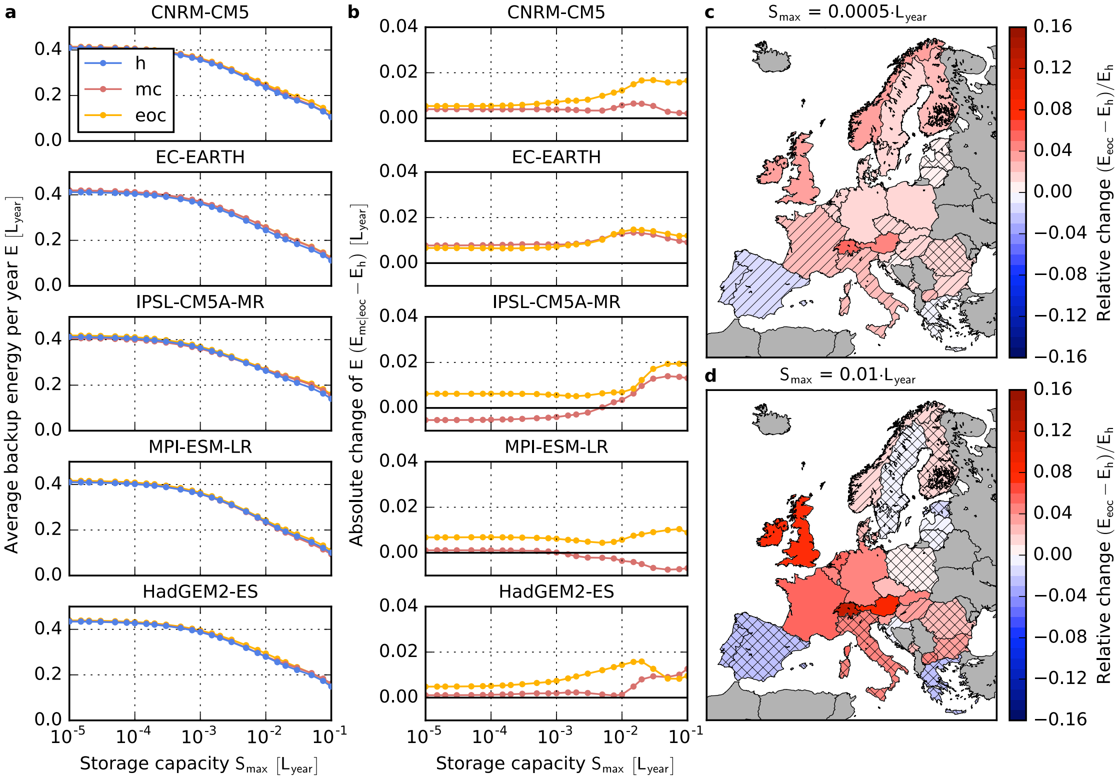

Fig A. Impact of medium climate change on backup energy needs in Europe. Parameters and presentation as in Fig 2.

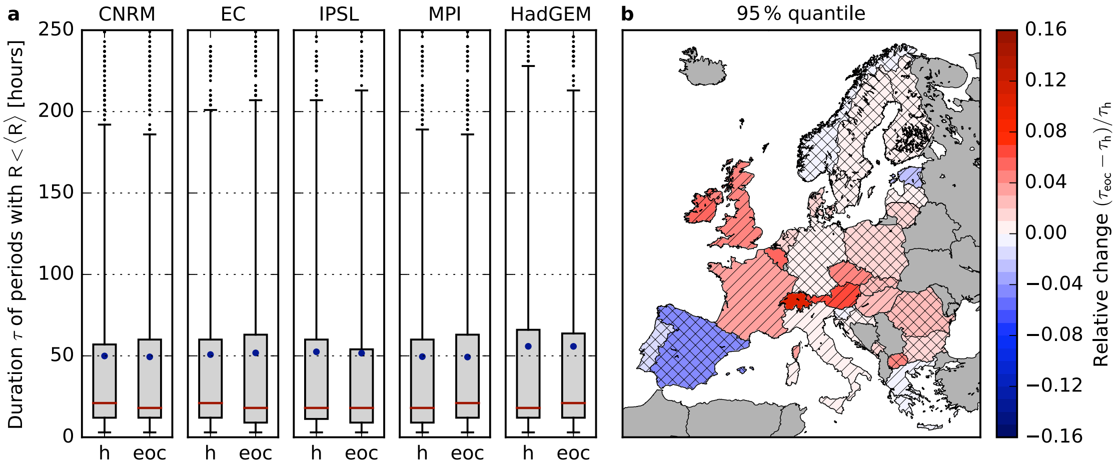

Fig B. Change of the duration of periods with low wind generation for a medium climate change scenario. Parameters and presentation as in Fig 5.

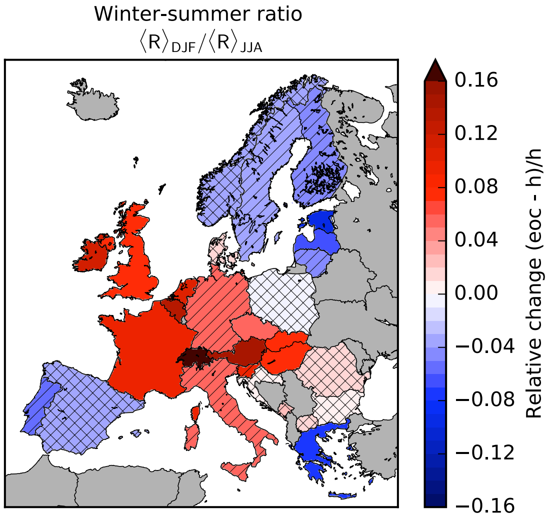

Fig C. Impact of medium climate change on the seasonal variability of wind power generation. Parameters and presentation as in Fig 7.

References

1. Van Vuuren DP, Edmonds J, Kainuma M, Riahi K, Thomson A, Hibbard K, et al. The representative concentration pathways: an overview. Climatic change. 2011;109:5–31.
