# Supplementary material for: Impact of climate change on backup energy and storage needs in wind-dominated power systems in Europe
Source: PLoS One. 2018 Aug 22;13(8):e0201457. doi: 10.1371/journal.pone.0201457 (PMC6104926; doi:10.1371/journal.pone.0201457)
Supplement: S4 Appendix — (PDF) [file pone.0201457.s004.pdf]

Appendix 4: Sensitivity studies

In order to examine the sensitivity of our system on different parameters, we repeated our analysis using

- different penetrations of renewables: $\gamma = 1.2$ and $\gamma = 0.8$, [1]
- a constant load [2],
- a different distribution of wind farms,
- a different hub height (120 m),
- a combination of wind and PV [1, 3–5].

Furthermore, we repeated the analysis of the CMIP5 ensemble by using one threshold value of the f-parameter (f_{th}) for all circulation weather types to characterize periods of low wind power generation.

Renewable penetration

Different penetrations of wind power are simulated by scaling the renewable generation using the γ factor: $\langle R \rangle = \gamma \langle L \rangle$. In the case $\gamma < 1$, on average an energy amount of $(1 - \gamma) \cdot L_{\text{year}}$ has to be provided by non-renewable power plants. Thus, we are interested in the additional backup energy $E_{\text{add}} = E - (1 - \gamma) \cdot L_{\text{year}}$ with $E = \langle B \rangle / \langle L \rangle \cdot L_{\text{year}}$.

In Figs A and B the change in the backup energy need is shown for $\gamma = 1.2$ and $\gamma = 0.8$, respectively. A comparison with Fig 2 of the main manuscript shows that absolute values of the backup energy (panels a) decrease (as expected). However, the absolute change of the backup energy (panels b) is quite similar and, thus, the relative change (panels c and d) is even higher for most countries (e.g. for UK the average relative change is 36.2 % for $\gamma = 0.8$ and 20.2 % for $\gamma = 1.2$ for $S_{\max} = 0.01 \cdot L_{\text{year}}$). For all countries, the average predicted sign of change is the same as for $\gamma = 1$. Less models agree an the sign of change only in the case of Spain, Greece and Croatia (only high $S_{\max}$) for $\gamma = 0.8$ compared to $\gamma = 1$.

Hence, a higher or lower renewable penetration also leads to increasing (decreasing) backup needs in many European countries, often even with higher relative changes.

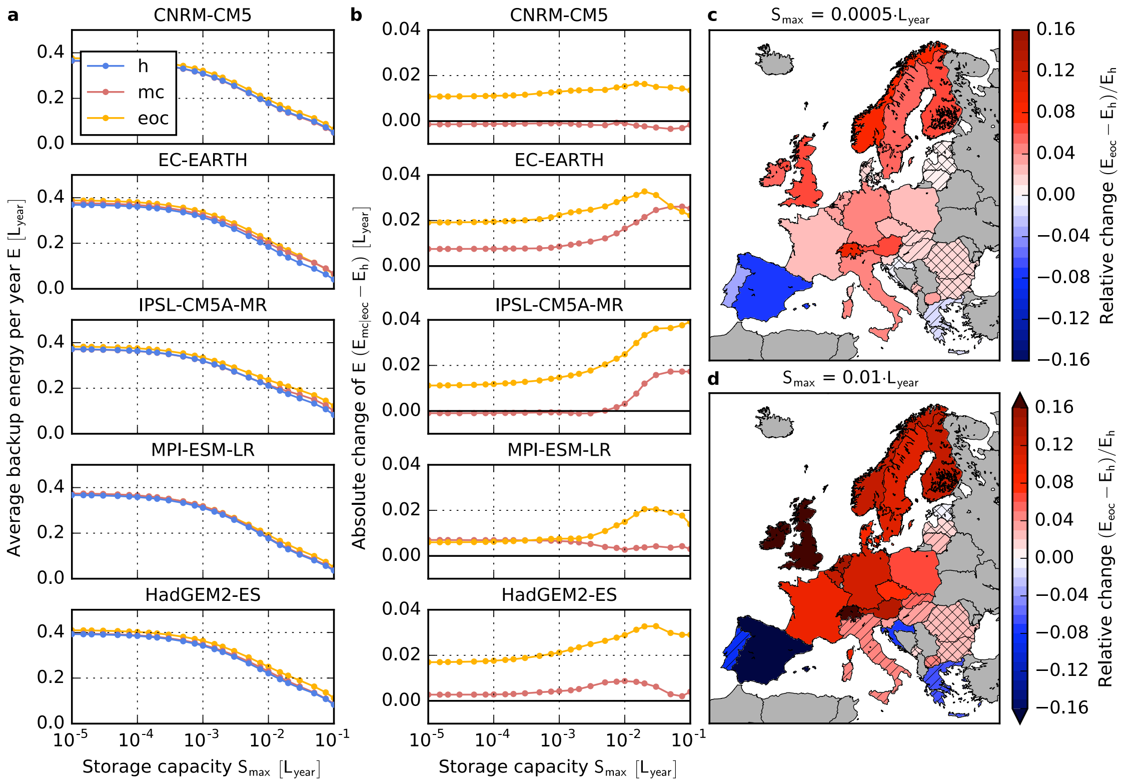

Fig A. Impact of strong climate change on backup energy needs in Europe for a renewable penetration of $\gamma = 1.2$. The maximum relative changes are 0.20 in Switzerland and -0.18 in Spain. Further parameters and presentation as in Fig 2.

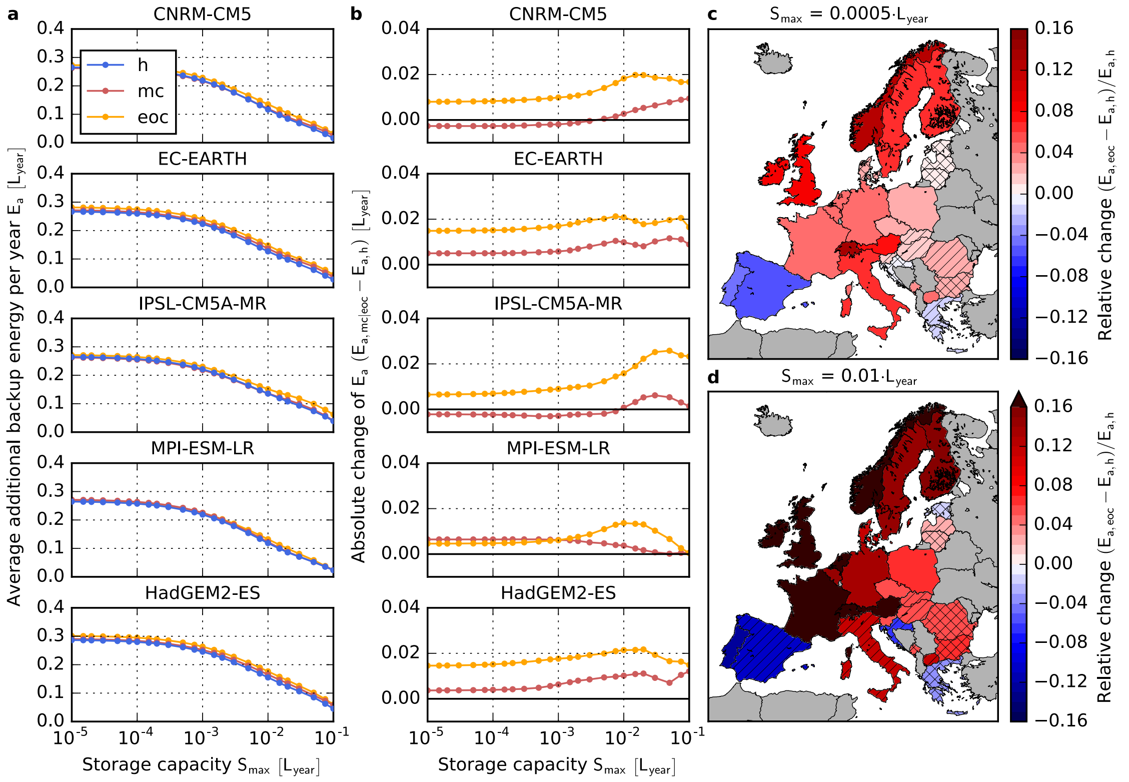

Fig B. Impact of strong climate change on backup energy needs in Europe for a renewable penetration of $\gamma = 0.8$. Shown is the amount of energy that has additionally to the pre-defined 20 % to be provided by dispatchable backup generators. The maximum relative change is 0.36 in UK. Further parameters and presentation as in Fig 2.

Different load time series

In order to analyze the sensitivity of the results on the applied load time series, we repeated our analysis using a constant value for the load such that: $L = \langle R \rangle$. The change of the backup energy need is shown in Fig C. A comparison with Fig 2 of the main manuscript reveals that the dependence of the results on the exact load time series is weak. Absolute values of the backup energy increase slightly (panels a). This comes from the fact that using a constant load leads to a loss of positive correlations: On average, there is more wind generation in winter than in summer. This correlates to a higher electricity demand in winter than in summer (in many countries). However, the relative change of the backup energy need is hardly affected. Only in Finland and Norway less models agree on the sign of change in the case of high $S_{\max}$.

Thus, we find only a weak dependence of the results on the exact load time series. This can further be explained by the fact that the fluctuations in the wind generation are much higher than fluctuations in the demand (cf. Fig 1c in the main manuscript) in scenarios with high wind penetrations.

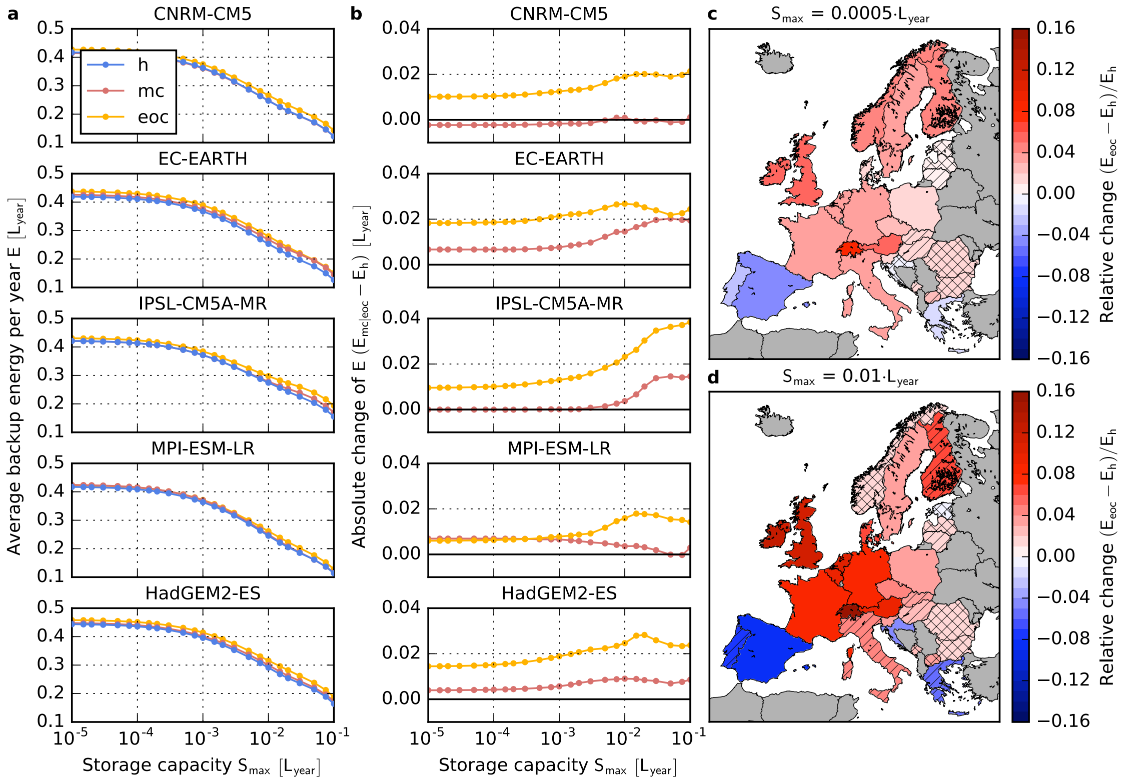

Fig C. Impact of strong climate change on backup energy needs in Europe for a constant load. Parameters and presentation as in Fig 2.

Different distribution of wind farms

In order to quantify the importance of the exact placement of wind farms, we repeated the analysis assuming a homogeneous distribution of wind farms at each grid point within a country.

A comparison of Fig D with Fig 2 of the main manuscript shows that absolute backup energy values slightly increase (panels a). This is because wind farms are sited on less windy locations on average. However, the relative change of the backup energy hardly depends on the exact wind farm distribution. For Portugal and Greece results become more robust (for high $S_{\max}$) whereas in Croatia results become less robust.

For Germany, the duration distribution of periods with $R(t) < \langle R \rangle$ is shifted to higher values for a homogeneous distribution of wind farms (cf. Fig E and Fig 5 in the main manuscript, panels a) because wind farms are sited less favorable. Regarding the relative change of the 95 % quantile of the duration distribution (panels b), results are less robust in Sweden, Austria, Estonia, Portugal and Slovenia whereas in Italy, Denmark, Greece and Latvia more models agree on the sign of change. In Italy, the sign of change flips – the duration of long periods with low wind power output tends to decrease. In all other countries, especially in Central Europe, France and the British Isles the qualitative results are the same.

The relative change of the winter-summer ratio is more pronounced if wind farms are placed homogeneously on each grid point within a country (cf. Fig F with Fig 7 in the main manuscript; be aware that the scales of the colorbars are different). The sign of change is the same in all countries. In Croatia and Slovakia results become less robust whereas in Finland and Lithuania more robust results are revealed.

All in all, the exact distribution of wind farms does hardly alter the results reported in this study (except for Italy).

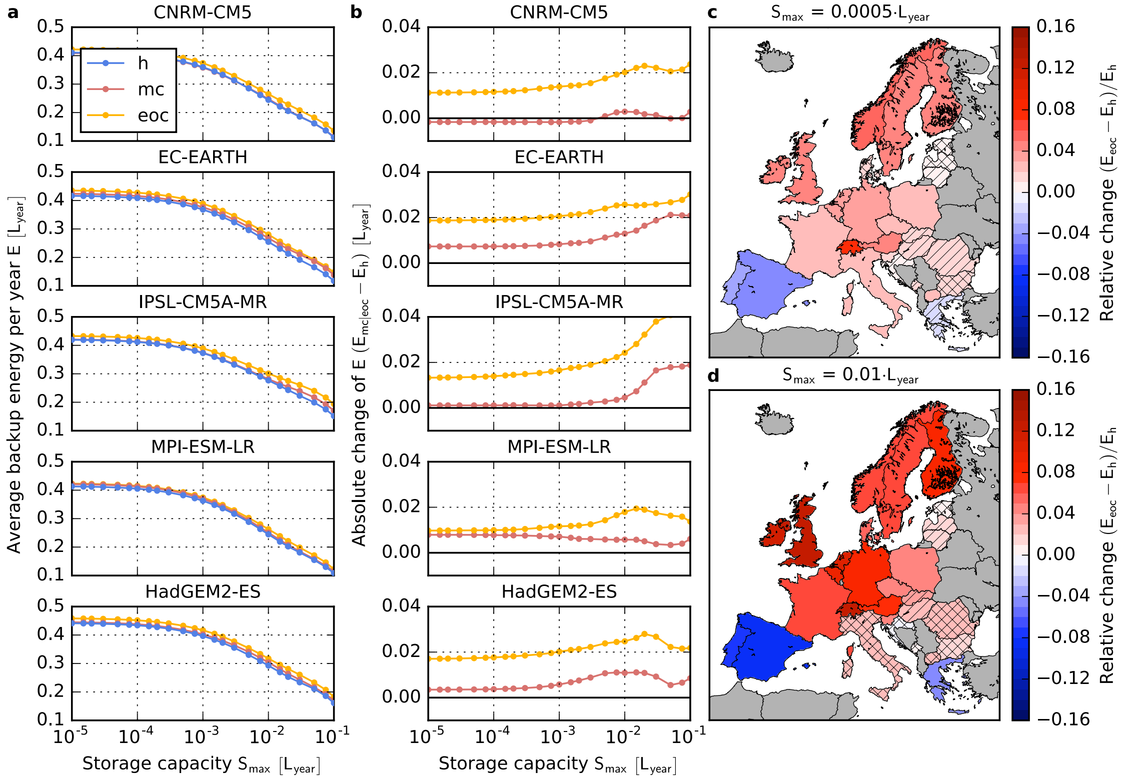

Fig D. Impact of strong climate change on backup energy needs in Europe for a homogeneous distribution of wind farms. Parameters and presentation as in Fig 2.

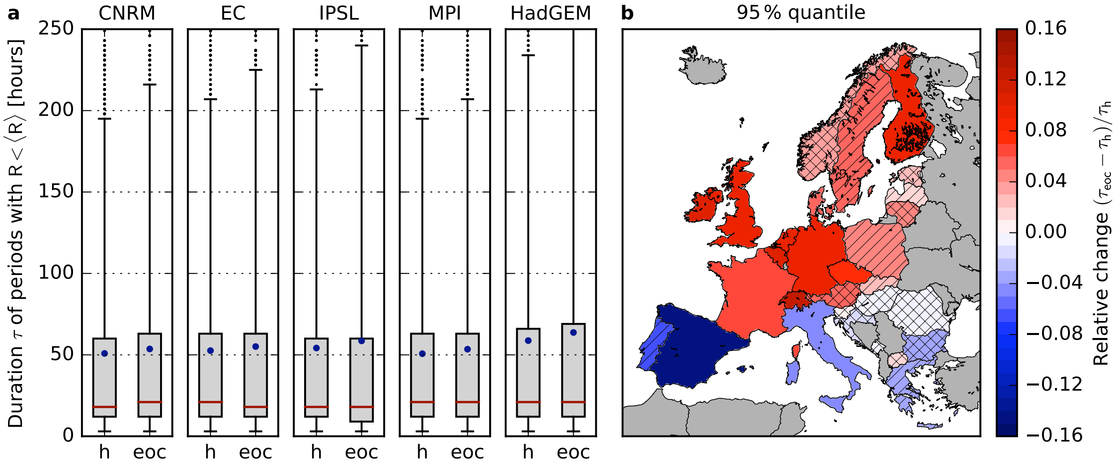

Fig E. Change of the duration of periods with low wind generation for a homogeneous distribution of wind farms. Parameters and presentation as in Fig 5.

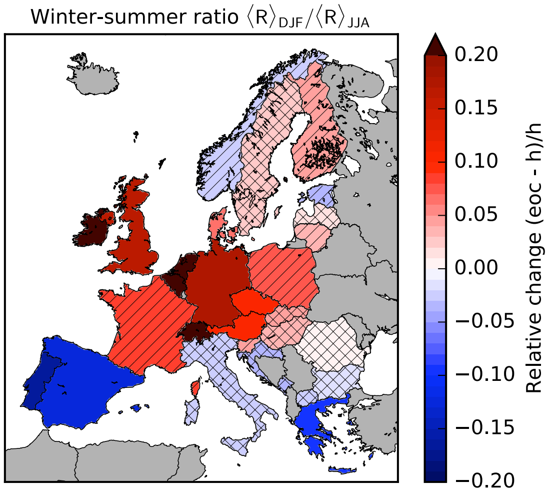

Fig F. Impact of strong climate change on the seasonal variability of wind power generation for a homogeneous distribution of wind farms. Parameters and presentation as in Fig 7. Be aware that the scale of the colorbar is different than in Fig 7.

Different hub height

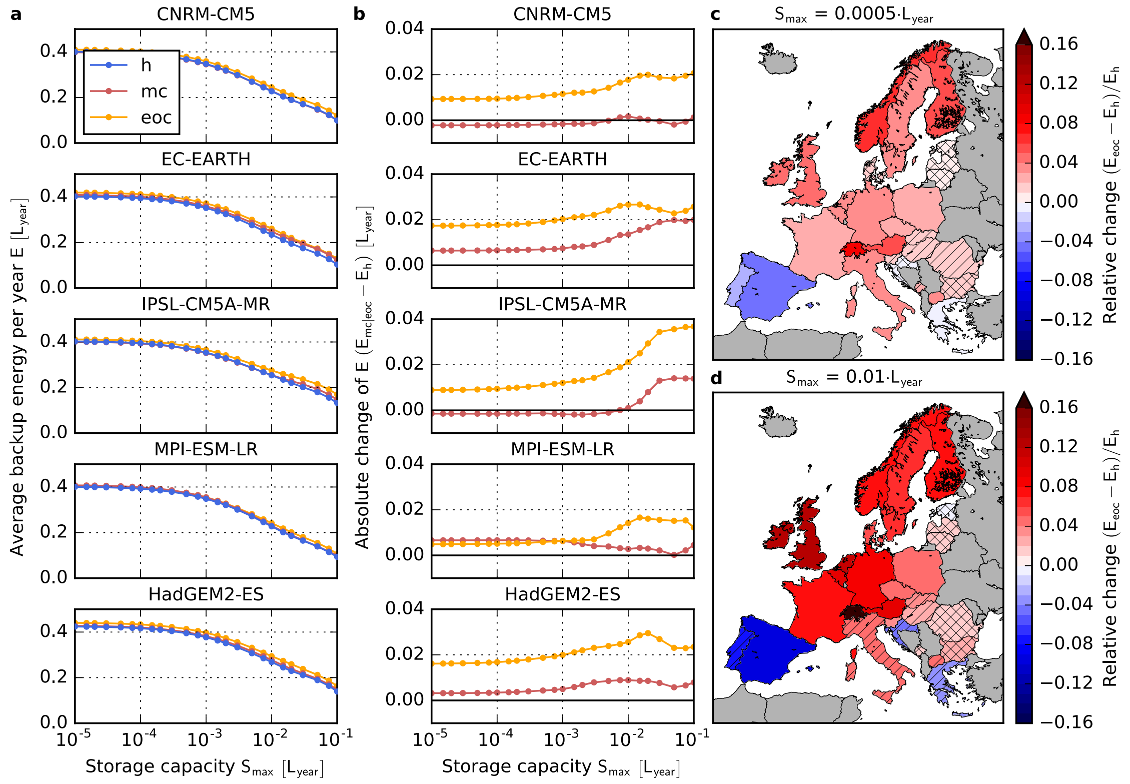

Fig G. Impact of strong climate change on backup energy needs in Europe at a hub height of 120 m Further parameters and presentation as in Fig 2.

In order to quantify the importance of the hub height of wind farms, we repeated the analysis using a hub height of 120 m.

A comparison of Fig. G with Fig. 2 of the main manuscript shows that absolute backup energy values slightly decrease (panels a). This is because wind farms experience higher wind speeds due to the higher hub height. However, the relative change of the backup energy hardly changes at all. Therefore, we conclude that the hub height plays only a very minor role in our analysis.

Wind and PV

We extend our analysis using a mix of wind and photovoltaics (PV) in order to evaluate the impact of PV on our results. For all scenarios and time frames we scale wind and solar photovoltaic (PV) power outputs such that they provide a fixed share γ of the load with a fixed mix α of wind and PV [3]. Hence, the renewable power generation can be adapted to

$$R(t) = R_{\mathrm{wind}}(t) + R_{\mathrm{PV}}(t) \tag{1}$$

with

$$R_{\mathrm{wind}}(t) = \gamma\, \alpha\, \frac{CF_{\mathrm{wind}}(t)}{\langle CF_{\mathrm{wind}} \rangle}\, \langle L \rangle, \tag{2}$$

$$R_{\mathrm{PV}}(t) = \gamma\, (1-\alpha)\, \frac{CF_{\mathrm{PV}}(t)}{\langle CF_{\mathrm{PV}} \rangle}\, \langle L \rangle. \tag{3}$$

CF_{wind} and CF_{PV} denote the country-wise aggregated capacity factors of wind and PV, respectively (as described in the main text for wind). The brackets denote the average over the respective time frame for a given model. Hence, $\langle R_{\mathrm{wind}} \rangle = \gamma\, \alpha\, \langle L \rangle$ and $\langle R_{\mathrm{PV}} \rangle = \gamma\, (1-\alpha)\, \langle L \rangle$, such that $\langle R \rangle = \gamma\, \langle L \rangle$.

We use PV-output data provided by the renewables.ninja data set (version v1.0) [5]. It consists of 30 years (1985-2014) of country-wise aggregated hourly PV capacity factors (i.e. generation per installed capacity) of Europe for the fleet of 2014 using MERRA-2 global meteorological reanalyses data. As we use a time frame of 31 years in our analysis, one year of data is repeated. Furthermore, the data is resorted such that leap years are matched. We do not use EURO-CORDEX output to calculate the PV generation because of the known shortcomings in the radiation budget in regional climate models due to cloud cover and albedo uncertainties [6] and because we want to focus here on changes in wind power generation.

In this sensitivity study, a mix of 70 % wind and 30 % PV (i.e. $\alpha = 0.7$) is simulated. This wind fraction is inspired by [4], who find an average optimal mix of $\alpha = 0.71$ for Europe.

It should be noted that due to this simplified procedure, the actual weather situations of the wind and the PV time series do not fit together. Furthermore, a potential change of the PV output due to climate change is not considered. However, as we are interested in changes of the temporal

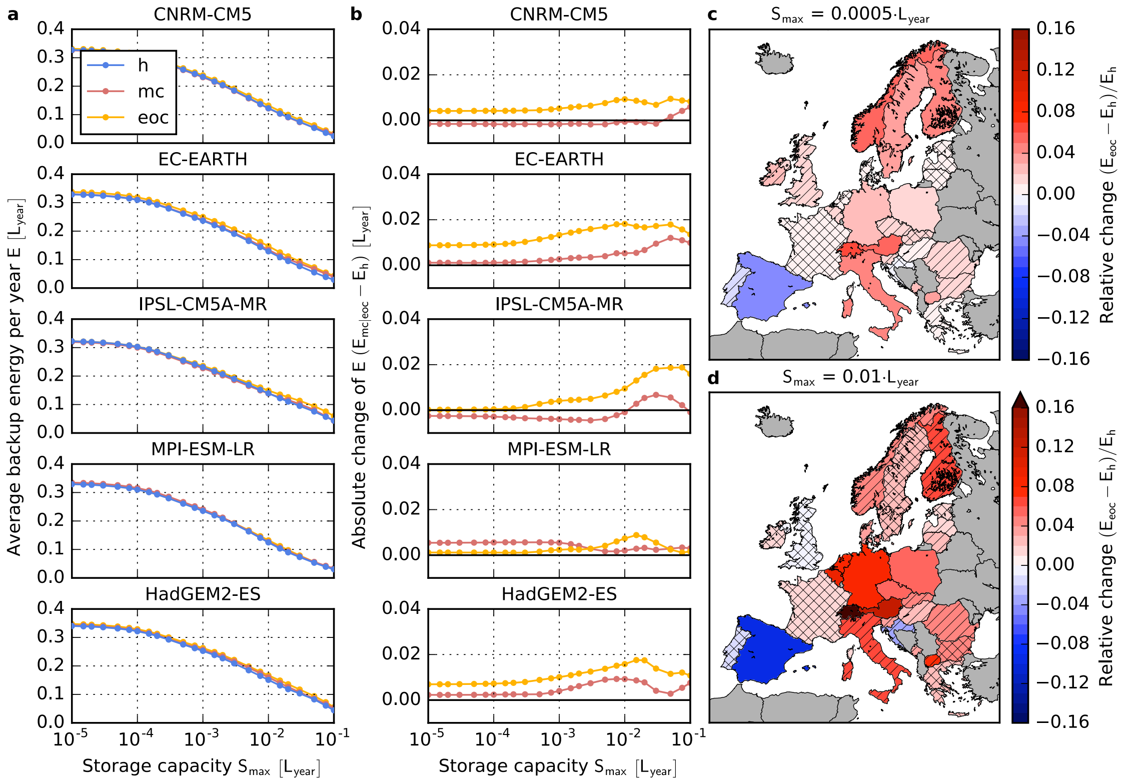

Fig H. Impact of strong climate change on backup energy needs in Europe for a wind-PV-mix with a wind fraction of $\alpha = 0.7$. Further parameters and presentation as in Fig 2.

characteristics of wind power generation, this estimate is sufficient for our studies.

The change in the backup energy need is shown in Fig. H. Compared to a pure wind scenario ($\alpha = 1$, cf. Fig. 2 of the main manuscript), absolute values of the backup energy decrease if a wind-PV-mix with $\alpha = 0.7$ is used (see panels a). This is because PV can compensate for the low wind power output in the summer months. However, relative changes are comparable to the pure wind scenario in most of Central Europe, Italy, Scandinavia, Spain and Croatia. For Scandinavia, the Czech Republic, the Netherlands, Belgium and Croatia, the robustness of the results depends on the storage size. Results are not robust for the British Isles, France, Portugal and Greece. Thus, on the British Isles and France, a mix of wind and PV may alleviate the impact of strong climate change on the backup energy need. For more detailed conclusions on this aspect, however, it would be necessary to study the exact impact of the wind fraction α on the results.

Repetition of the analysis of the CMIP5 ensemble using a different threshold value of the f-parameter

In the main manuscript, we determined the duration of periods with low wind generation by deriving one threshold value f_{th} for each CWT. It is important to note that the determined threshold value f_{th} is not perfect, as the distance $(1 - \mathrm{SEN})^2 + \mathrm{FFP}^2$ is far away from being zero (see red dot in Fig C in S1_Appendix). Hence, there is still a certain amount (for the western CWT: 19 %) of false predictions. Thus, the derived duration distribution depends on the choice of the threshold value f_{th}. To assess the sensitivity of the choice of f_{th}, we repeat our analysis by determining one value for f_{th} which is independent of the underlying CWT. The result is shown in Fig I. A comparison to Fig 8b in the main manuscript shows that in both cases the ensemble of the 22 GCMs predicts the same: The mean, the 90 % quantile and the 95 % quantile tend to increase by the end of the century. However, the value of a single model may be shifted for the 90 % quantile and the 95 % quantile: E.g. for HadGEM2-ES the absolute change of the duraton of scarcity of the 90 % quantile is one day in Fig 8b and two days in Fig I. An explanation for this is that already a slight shift of the threshold value f_{th} can split one long period with $f(t) \leq f_{\mathrm{th}}$ into two shorter periods with $f(t) \leq f_{\mathrm{th}}$ and a short period with $f(t) > f_{\mathrm{th}}$ in between or vice versa. Therefore, the results for the extremes (i.e. the 90 % quantile and the 95 % quantile) may be shifted for a single model. This effect averages out over the ensemble.

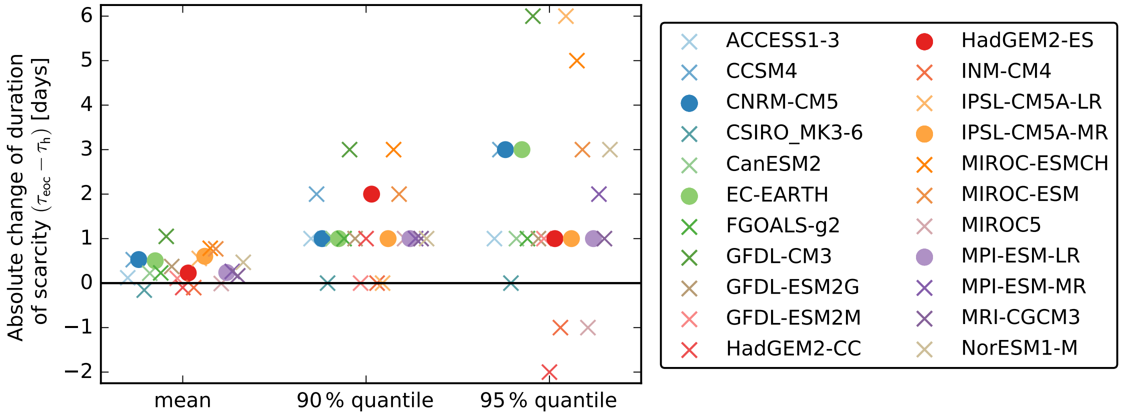

Fig I. Absolute change of the duration of periods with low wind generation using one $\mathbf{f_{th}}$ for all CWTs. Parameters and presentation as in Fig 8b.

References

1. Rasmussen MG, Andresen GB, Greiner M. Storage and balancing synergies in a fully or highly renewable pan-European power system. Energy Policy. 2012;51:642–651.

2. European Network of Transmission System Operators for Electricity (ENTSO-E). Hourly load values; accessed December 10, 2016. `https://www.entsoe.eu/db-query/consumption/mhlv-all-countries-for-a-specific-month`.

3. Heide D, von Bremen L, Greiner M, Hoffmann C, Speckmann M, Bofinger S. Seasonal optimal mix of wind and solar power in a future, highly renewable Europe. Renewable Energy. 2010;35:2483.

4. Rodriguez RA, Becker S, Andresen GB, Heide D, Greiner M. Transmission needs across a fully renewable European power system. Renewable Energy. 2014;63:467–476.

5. Pfenninger S, Staffell I. Long-term patterns of European PV output using 30 years of validated hourly reanalysis and satellite data. Energy. 2016;114:1251–1265.

6. Kothe S, Dobler A, Beck A, Ahrens B. The radiation budget in a regional climate model. Climate dynamics. 2011;36(5-6):1023–1036.
